# The effect of the community midwifery model on maternal and newborn health service utilization and outcomes in Busia County of Kenya: a quasi-experimental study

**DOI:** 10.1186/s12884-020-03405-w

**Published:** 2020-11-19

**Authors:** Duncan N. Shikuku, Geofrey Tanui, Mercy Wabomba, Dennis Wanjala, Josephine Friday, Taphroze Peru, Evelyne Atamba, Kenneth Sisimwo

**Affiliations:** 1 Save the Children, Busia, Kenya; 2 Department of Health, Busia, Kenya

**Keywords:** Community midwife, Skilled pregnancy and childbirth attendance, Maternal and newborn health care, Hard-to-reach communities, Rural Kenya

## Abstract

**Background:**

Poor women in hard-to-reach areas are least likely to receive healthcare and thus carry the burden of maternal and perinatal mortality from complications of childbirth. This study evaluated the effect of an enhanced community midwifery model on skilled attendance during pregnancy/childbirth as well as on maternal and perinatal outcomes against the backdrop of protracted healthcare workers’ strikes in rural Kenya.

**Methods:**

The study used a quasi-experimental (one-group pretest-posttest) design. The study spanned three time periods: December 2016-February 2017 when doctors were on strike (P1), March-May 2017 when no healthcare providers were on strike (P2), and June-October 2017 when nurses/midwives were on strike (P3), which was also the period when the project enhanced the capacity of community midwives (CMs) to provide services at the community level. Analysis entailed comparison of frequencies/means of maternal and newborn health service utilization data across the three periods.

**Results:**

The monthly average number of clients obtaining services from CMs across the three time periods was: first antenatal care (ANC) (P1-1.8, P2-2.3, P3-9.9), fourth ANC (P1-1.4, P2-1.0, P3-7.1), skilled birth (P1-1.5, P2-1.7, P3-13.1) and the differences in means were statistically significant (*p* < 0.05). Over the period, the monthly average number of clients obtaining services from health facilities was: first ANC (P1-55.7, P2-70.8, P3-4.0), fourth ANC (P1-29.6, P2-38.1, P3-1.2) and skilled birth (P1-63.1, P2-87.4, P3-5.6), *p* < 0.05. There were no statistically significant differences in the average number of clients obtaining services from CMs or health facilities between P1 and P2 (*p* > 0.05). There was, however, a statistically significant increase in the average number of clients obtaining services from CMs in P3 accompanied by a statistically significant decline in the average number of clients obtaining services from health facilities (*p* < 0.05). First ANC increased by 68%, fourth ANC by 75%, skilled births by 68%, and postnatal care by 33% in P3 (*p* < 0.0001). There was a non-significant decline in macerated stillbirths and neonatal deaths in P3.

**Conclusions:**

The findings underscore the importance of integrating community-level health service providers (CMs and health volunteers) into the primary health care system to complement service delivery according to their level of expertise, especially in low-resource settings.

## Background

Globally, the latest estimates indicate that 295,000 maternal deaths occurred in 2017, 2.5 million newborns died in 2018, and 2.6 million stillbirths occurred in 2015 with 99 percent, 77 percent and 98 percent of them occurring in low- and middle-income countries respectively [1–3]. The risk of a mother dying in a low- and middle-income country is 23 times higher than in a high income country (maternal mortality ratio (MMR) of 254 vs. 11 per 100,000 live births) with sub-Saharan Africa having the highest MMR at 534 per 100,000 live births compared to Europe and Central Asia with the lowest MMR at 11 per 100,000 live births [1]. Globally, an estimated 72 percent of births are now attended by a skilled health personnel (SHP) [4]. This however varies according to income group (46 percent in low-income groups and 99 percent in high-income groups) and by geographical area (48 percent in the African region and 99 percent in Europe) [4]. Poor women in remote areas are the least likely to receive adequate health care and carry the burden of maternal and neonatal morbidity and mortality related to complications of childbirth [5]. This clearly indicates that strategies and policies to address financial risk protection and access to quality essential healthcare services must be adopted to achieve universal health coverage [6]. These variations reflect inequities around the world in access to health services, highlight the disparities/gaps between countries, within countries, and between women with high and low income and those living in rural versus urban areas [7].

Healthcare solutions to prevent or manage complications of childbirth are well known. All women need access to antenatal care (ANC) in pregnancy, skilled care during childbirth, and care and support in the weeks after childbirth [7]. Countries are united behind Sustainable Development Goal 3 to reduce the global maternal mortality ratio to less than 70 per 100,000 live births, and to have no country with a maternal mortality rate of more than twice the global average [7]. To improve maternal health, barriers that limit access to quality maternal health services must be identified and addressed at all levels of the health system. As part of the Global Strategy and goal of Ending Preventable Maternal Mortality, the World Health Organization (WHO) supports countries through advocacy for more affordable and effective treatments, guidelines and policies towards addressing inequalities in access to and quality of reproductive, maternal, and newborn health care services and ensuring universal health coverage for comprehensive reproductive, maternal, and newborn health care [7]. Africa experiences a critical shortage of healthcare providers. The WHO reported that 36 of the 57 countries facing chronic human resource shortages in the health sector are in Sub – Saharan Africa [8] and that the ratio of nursing/midwifery personnel per 10,000 people in Africa (12.0 per 10,000) is nearly seven times lower than in Europe (80.5 per 10,000) [4]. The shortage of skilled birth attendants in Africa is even more severe in rural compared to urban areas [4].

Family planning and maternal and newborn health interventions delivered through the midwifery model could avert a total of 83 percent of all maternal deaths, stillbirths and neonatal deaths [9]. Midwives are skilled health personnel (SHP) providing care during childbirth. WHO, International Federation of Gynecology and Obstetrics (FIGO) and International Confederation of Midwives (ICM) define a SHP as a competent maternal and newborn health (MNH) professional educated, trained and regulated to national and international standards competent to: provide and promote evidence-based, human-rights-based, quality, socio-culturally sensitive and dignified care to women and newborns; facilitate physiological processes during labour and delivery to ensure a clean and positive childbirth experience; and identify and manage or refer women and/or newborns with complications [10]. Within an enabling environment (one that provides supportive regulation, policies and infrastructure, communication, referral, logistics, and supplies, inputs that are necessary for a skilled attendant to provide care) [11], midwives can provide 87 percent of the needed essential care for women and newborns, when educated and regulated to international standards [12]. To achieve this, midwives must be equitably distributed, accessible by the population and possess the required competencies and motivation to deliver quality care that is appropriate and acceptable to the sociocultural contexts and expectations of the served population [13].

Evidence around the world (especially in Sri Lanka and Pakistan) including Kenya’s rural and peri – urban areas has shown that community – based interventions (including community midwives) improve ANC coverage, intrapartum care and reproductive health/family planning, maternal and neonatal health care and outcomes for the poor and disadvantaged women [11, 14–21]. The World Health Organization specially emphasizes the role of community midwives (CMs) and other community – based interventions in promoting safe motherhood particularly in rural settings with low access to health services [22, 23]. However, lack of skills – both clinical and entrepreneurial, and access to funds necessary to develop their practice infrastructure and logistics are major bottlenecks in their operations [11, 20, 21, 24, 25].

Kenya’s MMR is estimated at 342 maternal deaths per 100,000 live births, which is higher than the global SDG target of less than 70 per 100,000 live births [1, 6]. Estimates from the 2014 Kenya Demographic and Health Survey showed that only 62 percent of births were assisted by a SHP [26]. Half of the women residing in rural areas are not attended to by a SHP [26]. A 2017 report on the first Confidential Enquiry into Maternal Deaths in the country showed that delays in reaching a health facility account for 42 percent of the maternal deaths. In addition, one in every five maternal deaths resulted from avoidable community-level factors [27]. This underscores the importance of community-based interventions for mproving maternal and perinatal outcomes in the country. Kenya commissioned the community midwifery model in 2006 in order to increase access to skilled attendance at birth. The first implementation guidelines were developed in 2007 and were revised in 2012 to address key policies outlined in the Kenya Health Policy (2012–2030) regarding the provision of essential packages for health, Vision 2030 (a national development blueprint) and the Community Health Strategy [28]. The guidelines were revised to standardize the implementation of community midwifery services as a strategy for improving skilled attendance in the provision of maternal and newborn health care at the community level. They highlighted the critical role of the community midwife in the provision of continuum of care during normal pregnancy, childbirth, postpartum period, and in counselling for and providing family planning services as well as newborn care and referral.

Two national industrial strikes by SHP (December 2016 – February 2017 and June – October 2017) crippled the country’s healthcare system and led to many deaths [29, 30]. During the second strike, Save the Children supported the Busia County Department of Health to implement an enhanced community midwifery model through the Boresha Project (April 2015-March 2018) funded by the United Kingdom’s Department for International Development (DfID). The project was a community-based intervention whose aim was to create demand, improve access and utilization of skilled antenatal, childbirth and postnatal care services in rural ‘hard-to-reach’ communities. The project used a three-pronged approach that targeted demand creation in the community for MNH services through the community health strategy; service delivery of MNH services in the health facilities focusing on quality improvement of MNH services through maternal and perinatal deaths surveillance and response; and advocacy to increase government commitment and targeted resources allocation to health. These interventions are essential in reducing preventable maternal and newborn mortalities in communities with low/reduced access to quality maternal and newborn health services. This study evaluated the effect of the enhanced community midwifery model on skilled attendance during pregnancy and childbirth and maternal and newborn health outcomes against the backdrop of a national strike by skilled health personnel in the public sector. The secondary objective was to review the effect of the national doctors and nurses/midwives’ strikes on availability and utilization of MNH services in rural settings of Busia County, Kenya.

## Methods

### Setting

Busia County has seven sub-counties and is predominantly a rural setting. The proportion of births in the county attended to by skilled health personnel is 59% (lower than the national average of 62%) [31]. In addition, the county has traditional birth attendants who provided home antenatal and delivery services. During the project, these were reoriented to become birth companions (BCs) and primarily provided support in health education and referral of pregnant women to the health facilities for skilled antenatal, birth and postnatal care. The project supported four rural hard-to-reach sub-counties with poor maternal and newborn health indicators: Teso North, Teso South, Nambale and Samia. In this study, ‘hard-to-reach’ refers to populations who have limited regular contact with skilled pregnancy and childbirth services including people living in areas ‘too far’ from health services. ‘Too far’ not only refers to the physical distance but also limited logistics and human resource capacity [32].

The Community Midwifery Model (CMM) in Kenya uses skilled out of work or retired licensed healthcare professionals who are resident within a given community and seeks to contribute towards the achievement of SDG 3 [28] by addressing the three delays that commonly contribute to maternal and perinatal mortality [33]. The three delays are (1) delay in deciding to seek appropriate care, (2) delay in reaching an appropriate health care facility and (3) delay in receiving adequate emergency care at the facility [34]. The primary role of community midwives is provision of a continuum of care during normal pregnancy, childbirth, postpartum period, and in counselling for and providing family planning services as well as newborn care and referral. To achieve this, they link with community health volunteers (CHVs), BCs, community health extension workers (CHEWs), local committees, facility staff and county teams to promote safe motherhood in the community. All the community midwives included in this study met the requirements for providing services as stipulated in the national guidelines: were retired health professionals (nurse/midwives) with midwifery skills and registered with the national regulator Nursing Council of Kenya; had valid practicing licenses (evidence of retention on a professional register with the Nursing Council of Kenya) and were residents in the communities they served [28]. Their activities were supervised by the respective sub-county health management teams.

Save the Children, in collaboration with the Busia County Department of Health, facilitated the work of the community midwives by supporting their training in emergency obstetrics and newborn care (EmONC) to enhance their skills in management of basic EmONC signal functions as well as providing the necessary logistics to enable them provide services (Table 1). The seven basic EmONC signal functions are: (1) administration of parenteral antibiotics, (2) administration of uterotonic drugs, (3) administration of parenteral anticonvulsants for pre-eclampsia and eclampsia (magnesium sulphate), (4) manual removal of retained placenta, (5) removal of retained products of conception (e.g., manual vacuum aspiration), (6) assisted vaginal delivery (vacuum extraction) and (7) neonatal resuscitation (with bag and mask) [35]. The health facilities to which the community midwives were linked provided them with pharmaceuticals (Oxytocin for prevention of postpartum hemorrhage) and non-pharmaceuticals supplies – gloves, syringes and needles, cotton wool and gauze. The project team and the respective sub-county health management teams (sub-county nursing officer, sub-county reproductive health coordinator and sub-county community strategy focal person) provided structured monthly and/or appropriate support supervision and mentorship on quality service delivery.

**Table 1:** Support package provided to community midwives

Support provided	Supported by
5-days training in emergency obstetrics and newborn care (EmONC) using national EmONC training curriculum	Save the Children
Antenatal care equipment: weighing scale – adult and infant; fetal scopes	Save the Children
Intrapartum care equipment: vaginal examination kits; delivery kits, partographs	Save the Children
Newborn resuscitation equipment – bag and mask/ambubag for ventilation (size 0 and 1) and penguin suction devices; baby outfits (sweater, cap/hat and socks)	Save the Children
Infection prevention & control decontamination and waste segregation buckets^a^	Save the Children
Antepartum, intrapartum and postpartum care job aids/protocols	Save the Children
Service delivery registers - mother and child health booklets, antenatal care registers, delivery registers; postnatal care registers	Sub-county health management team
Referral forms and registers and referral telephone numbers	Sub-county health management team

^a^Where sterilizing equipment were not available, link health facilities supported this function as appropriate

### Scope of community midwifery services

The community midwives provided a range of antenatal, delivery and postnatal care services as recommended by the national guidelines in provision of community midwifery [28] and health services [36] (see Table 2). Importantly, all the childbirth services were conducted at the community midwife’s clinic and in exceptional cases, for instance, insecurity (especially at night), long distances and requests from clients, some were conducted at the community midwives’ homes, which was consistent with findings from an evaluation of the model in the country [37].

**Table 2:** Services offered by the community midwives

Period	Services offered
Antenatal	Dissemination of key messages on danger signs in pregnancy, birth planning and emergency preparedness to support safe pregnancy and delivery of a healthy newborn and early childhood care; monitoring and assessment of pregnancy through focused antenatal care (FANC) model; intermittent preventive treatment for malaria in pregnant women (IPT); tetanus toxoid vaccination; referral for antenatal profile; counselling and testing for HIV among pregnant women^a^
Childbirth	Childbirth care in uncomplicated labour and delivery (Essential Obstetric Care); provision of EmONC signal functions^b^; stabilizing women and/or their newborns who have complications prior to referral;
Newborn	Provision of essential newborn care – warmth, resuscitation, early initiation of breast feeding, nutritional counselling & hygiene
Postnatal	Targeted health education/information on danger signs, early detection and treatment of problems, care of breasts, advise on caring for the newborn; immunizations as per the Kenya Expanded Program on Immunization schedule, counselling and testing for HIV among the pregnant and postnatal mothers and provision of family planning counselling and services
Family planning	Provision of family planning counselling and methods – pills, injectables, implants and intra-uterine contraceptive devices

^a^HIV counselling available but testing not available in all the clinics

^b^EmONC signal functions provided include administration of parenteral oxytocics, administration of parenteral antibiotics, administration of parenteral anticonvulsants, manual removal of placenta and newborn resuscitation

Community midwives admitted clients for childbirth services. The period of stay at the CM varied from 1 to 3 days depending on the condition of the mother. Cases that required further review and care were referred to the nearby private facility to prevent obstetric complications. There was a minimal user fee for services provided by the CMs. However, the CMs did not deny women services for lack of payment in line with the universal health coverage policies. Non-monetary items and/or gifts in kind were also a form of payment that was acknowledged by the CMs for the services rendered to the community. In a few occasions however, the services were provided for free depending on the client’s socio – economic status. The project supported a reimbursement of KSh. 100 ($1) for BCs for every appropriate referral for a pregnancy, childbirth or postpartum condition with danger signs to the CMs. The danger signs for referral included maternal – vaginal bleeding, reduced or lack of movements of the unborn baby, convulsions, pale, fever, severe headache and severe abdominal pain; neonatal – refusal or poor breastfeeding, infection/fever, convulsions and difficulty breathing.

### Study design

The study used a quasi-experimental (one-group pre- and post-test) design. The aim was to determine the effect of the intervention (an enhanced community midwifery model) on maternal and newborn health service utilization and outcomes among hard-to-reach communities against the backdrop of the protracted healthcare workers’ strikes. The intervention was implemented over a period of 11 months from December 2016 to October 2017. In the first 6-month period, midwifery services were less affected in the public health sector health facilities (during a doctors’ strike in the first 3 months followed by a 3-month return to normalcy) compared to the last 5-month period (when the enhanced community midwifery model was strengthened due to nurses/midwives’ industrial action – see separate section describing intervention in detail below). To determine the effect of the enhanced community midwifery model, we aggregated periods 1 and 2 (first 6 months) into pre-intervention and considered period 3 (last 5 months) as post-intervention period.

**Fig. 1:**
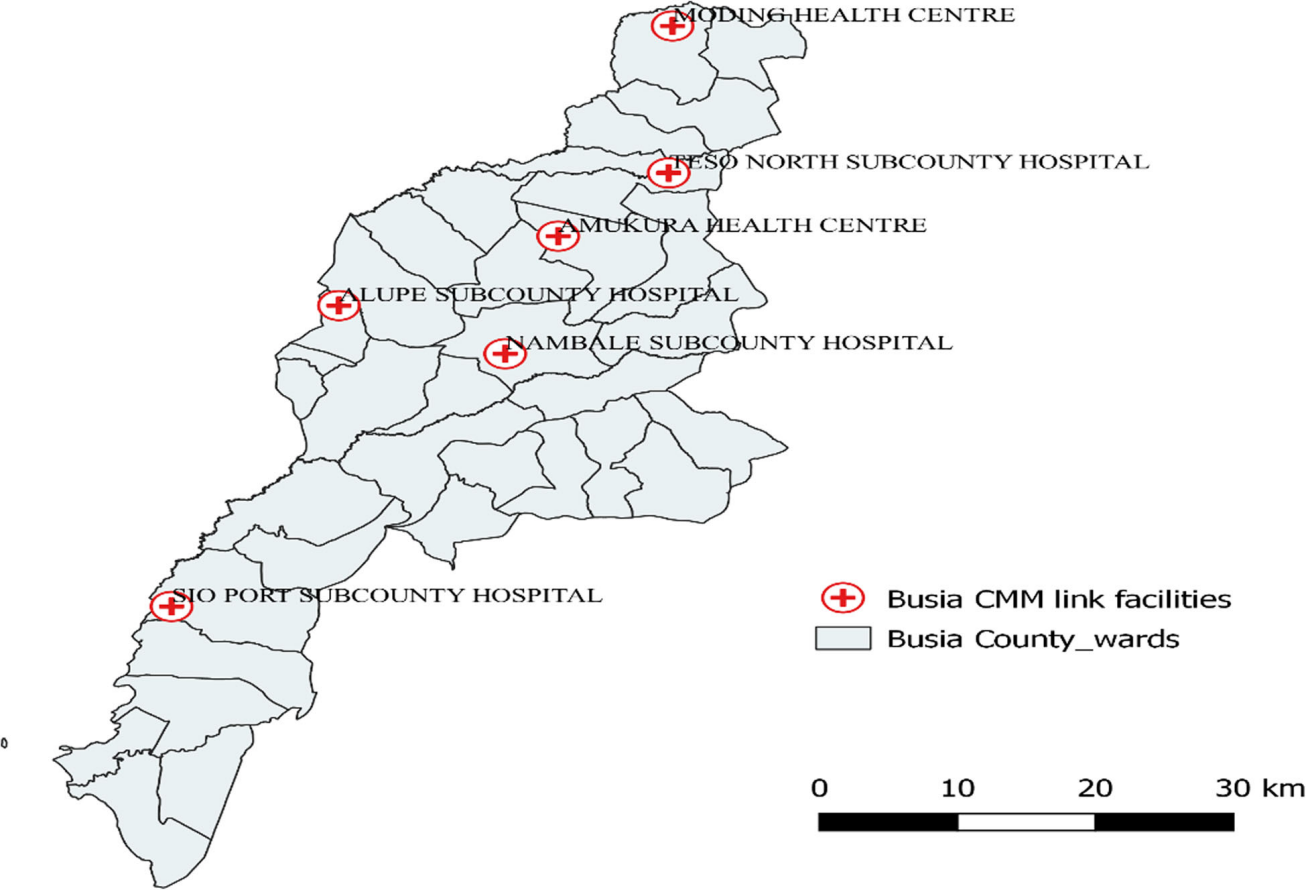
Map showing health facilities to which community midwives are linked in Busia County. *(Authors’ own; generated using QGIS software)

The study involved community midwives linked to six health facilities in Busia County. The health facilities were two comprehensive EmONC (Teso North sub-county hospital and Alupe sub-county hospital) and four basic EmONC (Sio Port sub-county hospital, Nambale sub-county hospital, Amukura health centre and Moding health centre) (Fig. 1).

### Intervention

The project introduced the community midwifery model in December 2016. During its implementation, there were two periods of industrial action by healthcare workers in the public sector over disagreements regarding terms of service: 3-month doctors’ strike between December 2016 – February 2017 followed by a 3-month period of return to normal services between March – May 2017 before the second 5-month nurses/midwives’ strike between June – October 2017. The project implemented an enhanced community midwifery model in the four sub-counties to improve skilled attendance during pregnancy and childbirth during the nurses/midwives’ strike.

A total of 10 community midwives were purposively identified across the four sub-counties included in the project and enrolled in the interventions. They were then linked to the nearby health facilities with high volumes of clients in their respective sub-counties. The community midwives were distributed as follows: one in Samia sub-county – linked to Sio Port Sub-county Hospital; three in Nambale sub-county – all linked to Nambale Sub-county Hospital; three in Teso North sub-county – two linked to Teso North Sub-county Hospital and one linked to Moding Health Centre; and three in Teso South sub-county – two linked to Amukura Health Centre and one linked to Alupe Sub-county Hospital.

**Fig. 2:**
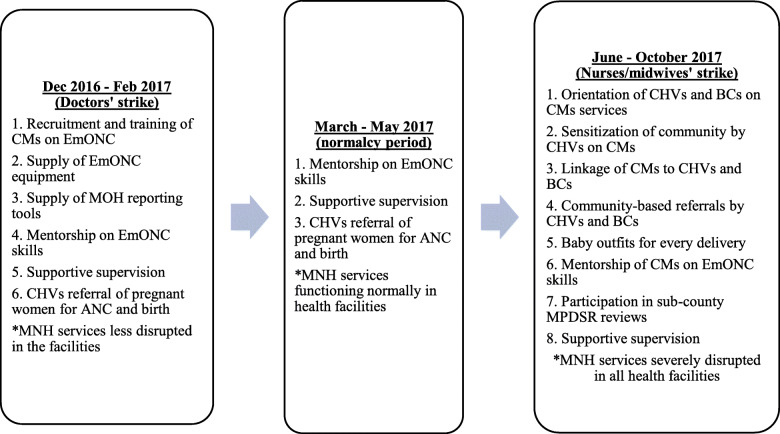
Chronology of intervention activities

During the doctors’ strike, maternal and newborn care service delivery (ANC, childbirth and postnatal care) were less affected in health facilities. During the nurses/midwives’ strike, maternal and newborn care services were severely affected across the country. During this time, the project supported a constellation of activities to create awareness and demand for community midwifery services. The activities included community sensitization by community health volunteers (CHVs) on danger signs in pregnancy and importance of seeking skilled antenatal, childbirth and postnatal care; linkage of the CMs with the CHVs; reorientation of traditional birth attendants (TBAs) to BCs – initiated during the strike by doctors; and linkage of the CMs and the CHVs with the reoriented BCs. Community midwives worked closely with CHEWs and CHVs in the provision of various health services at the community level; collection and reporting of monthly service utilization data as well as participation in the sub-county quarterly maternal and perinatal deaths surveillance and response (MPDSR) review meetings. In addition, the project team and the sub-county reproductive health and community health teams conducted support supervision and mentorship of CMs on emergency obstetrics and newborn care skills. The CHVs and BCs encouraged and referred pregnant women in their catchment areas to the nearby CM for antenatal, childbirth and postnatal care (Fig. 2).

### Data collection

The project used the Kenya Ministry of Health (MOH) reporting tools that health facilities, community midwives and community health volunteers use to capture information on service utilization. Data on the numbers of pregnant women seeking antenatal and birth services from facilities and community midwives were collected using the monthly MOH 711 summary report (Integrated Summary Report: Reproductive & Child Health, Medical and Rehabilitative Services). This is a secondary reporting tool with all the ANC and maternity service utilization data summarized from the primary daily activity reporting registers and is open – access and publicly available on the DHIS2 – the national MOH reporting system for use by health facilities, sub-counties and counties. The sub-county reproductive health and the records and information management teams verified the data in the primary MOH tools: ANC register (MOH 405), maternity register (MOH 333) and postpartum care register (MOH 406). First and fourth ANC visits were used to determine access and utilization of focused ANC services respectively as recommended [38]. The maternity register captured data on skilled births while the postnatal register captured information on utilization of postnatal care services for the mothers who had either received delivery services at the CMs and/or had unskilled home births and sought skilled post-delivery care or immunization.

Services provided by CHVs (referrals for ANC and skilled birth) were captured on MOH 514 form (Community Health Service Delivery Logbook) while CHEWs used MOH 515 form (Community Health Extension Worker Summary) to summarize information from MOH 514 forms. Community health extension workers and facility-based health care providers verified data from MOH 514 during routine monthly review meetings with CHVs before being uploaded on to MOH 515 in DHIS2. Data capture for the reporting month in the DHIS2 is completed by the 15th day of the subsequent month. Final data for this study were accessed from the DHIS2 on 6th April 2020.

### Variables and measurements

We analysed service utilization data for first and fourth ANC, birth and postpartum care provided by facilities and CMs for the periods of interest. A skilled birth was defined as a birth attended to by skilled health personnel - including a doctor, nurse or midwife - who is educated and trained to proficiency in the skills needed to manage normal (uncomplicated) pregnancies, childbirth and the immediate postnatal period, and in the identification, management and referral of complications in women and newborn babies [10, 39]. Postnatal care was defined as care to a woman 2–3 days post-delivery by a skilled health personnel – either in the health facility or by a community midwife.

### Data analysis

First author extracted raw data from the DHIS2, entered in Microsoft Office Excel 2013, cleaned and exported to STATA version 12 for analysis. The first author extracted the data from DHIS 2 in Excel format, cleaned and exported it to STATA version 12 for analysis. Service utilization data were classified into three periods or groups. The three periods of analysis included the three-month strike by doctors between December 2016 and February 2017 (Period 1), the three-month period of normalcy following the end of the doctors’ strike (Period 2 occurring between March and May 2017), and the five-month strike by nurses/midwives between June and October 2017 (Period 3). We computed the performance mean scores of outcomes of interest (1st ANC attendance, 4th ANC attendance, skilled births and postnatal care) for the three periods. We conducted one-way analysis of variance (ANOVA) to test for significant differences in the mean scores of outcomes of interest between time periods [40]. We also conducted Kruskal-Wallis test for distributions with small numbers [41]. We further conducted Tukey post hoc tests to determine which time periods were significantly different from each other in terms of maternal and newborn health service utilization and outcomes. The results are reported as effect sizes with 95% confidence intervals.

To determine the effect of the community midwifery model, we aggregated periods 1 and 2 into pre-intervention and considered period 3 as post-intervention period. We calculated indicators of performance by community midwives by comparing the number of antenatal care clients and deliveries conducted by this cadre as a fraction of the total number of antenatal care clients and deliveries conducted by health facilities and community midwives. We conducted two-groups test of proportions. Estimates with p-values less than 0.05 (*p* < 0.05) were considered statistically significant.

## Results

### Level of maternal and newborn health service utilization

**Fig. 3:**
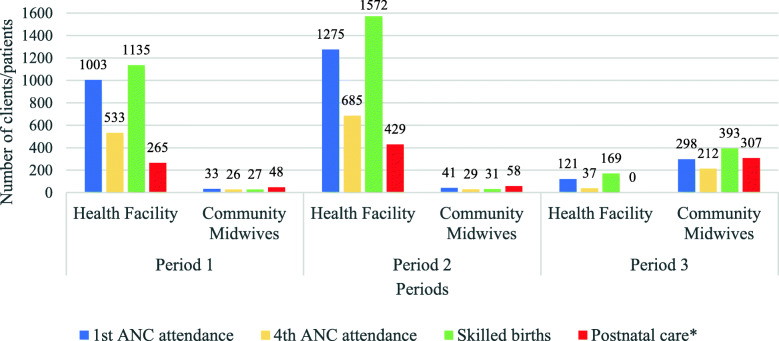
Maternal and newborn health service utilization by period and type of provider

The number of clients for first and fourth ANC and skilled births served by community midwives was high during period 3 when nurses/midwives were on strike compared to the previous two periods, including Period 1 when doctors were on strike. Health facility attendance was also lowest during the strike by nurses/midwives in period 3 compared with the other two periods (Fig. 3).

### Maternal and newborn health services at the community level

**Fig. 4:**
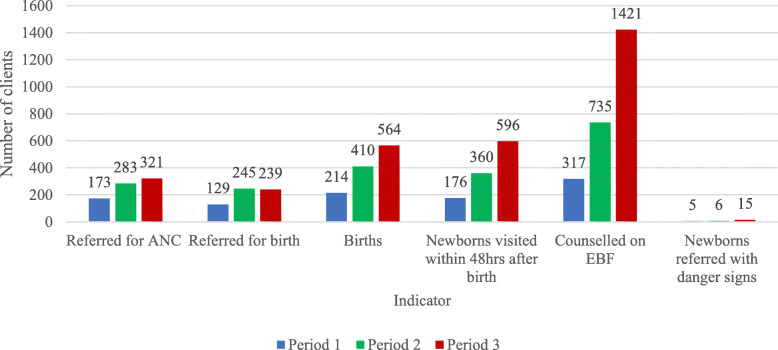
Maternal and newborn health service utilization at the community level by type of service and time period

Community health volunteers continued to provide health education to the community on maternal and newborn health care services in all the three periods. There was progressive increase in the number of clients referred by CHVs for ANC, births, newborns visited within 48 hours after birth, postnatal mothers counselled on exclusive breastfeeding (EBF) and newborns identified with danger signs referred for hospital care over time. Trends for unskilled community births also progressively increased across the three periods (Fig. 4).

### Means scores for maternal and newborn health service utilization

The overall mean scores for 1st and 4th ANC, skilled births and postnatal care utilization at the health facility level were 43.5 (± 37.5), 23.0 (± 20.3), 52.0 (± 48.2) and 28.9 (± 22.5) respectively with 4th ANC attendance having the lowest and skilled births the highest mean scores compared with the other services. On average, the number of women who sought first and fourth antenatal care, skilled births and postnatal care services from health facilities was highest during period 2 (when there was no strike) and lowest during period 3 (during the strike by nurses/midwives). At the community midwifery units, the overall means for 1st ANC, 4th ANC, skilled births and postnatal care were 4.7 (± 5.6), 3.2 (± 4.5), 5.4 (± 6.3) and 5.4 (± 8.7) respectively. On average, the number of women who sought first antenatal care, skilled births and postnatal care services from community midwives was lowest during period 1 (during the strike by doctors) and highest during period 3 (during the strike by nurses/midwives). Fourth antenatal care utilization was highest during period 3 (during the strike by nurses/midwives) and lowest during period 2 (when there was no strike). At both the health facility and community midwifery units, there were significant differences between the three periods for 1st ANC, 4th ANC and skilled birth attendance (*P* ≤ 0.05). For community maternal and newborn health services, the mean scores for clients referred for ANC and skilled births, community births and newborn visitation within 48 hours after birth were higher during period 2 (normalcy period) compared to the other periods although there were no significant differences between the three periods (Table 3).

**Table 3:** Mean scores for maternal and newborn health service utilization at facility, community midwifery and community units

	Total	Period 1	Period 2	Period 3	ANOVA	*P*-value
	Mean (SD)	Mean (SD)	Mean (SD)	Mean (SD)	F-value	
Facility level performance						
1st ANC attendance	43.5 (37.5)	55.7 (21.7)	70.8 (36.6)	4.0 (4.2)	12.05	0.0008*
4th ANC attendance	23.0 (20.3)	29.6 (14.0)	38.1 (17.7)	1.2 (1.5)	13.15	0.0005*
Skilled births	52.0 (48.2)	63.1 (35.0)	87.4 (49.0)	5.6 (6.6)	8.66	0.0032*
Postnatal care**	28.9 (22.5)	22.1 (12.9)	35.8 (29.8)	**		0.2160
Community midwives' performance						
1st ANC attendance	4.7 (5.6)	1.8 (1.2)	2.3 (1.5)	9.9 (7.3)	6.62	0.0087*
4th ANC attendance	3.2 (4.5)	1.4 (1.0)	1.0 (0.8)	7.1 (6.3)	5.04	0.0212*
Skilled births	5.4 (6.3)	1.5 (1.3)	1.7 (1.5)	13.1 (5.2)	26.03	<0.0001*
Postnatal care	5.4 (8.7)	2.7 (2.4)	3.2 (2.9)	10.2 (14.1)	1.5	0.2543
CHEW level performance						
Referred for ANC	12.0 (10.0)	9.6 (10.0)	15.7 (10.8)	10.7 (10.0)	0.6	0.5598
Referred for birth	9.6 (7.5)	7.2 (5.8)	13.6 (10.0)	8.0 (5.5)	1.36	0.2869
Births (community)	17.8 (9.3)	11.9 (6.2)	22.8 (11.4)	18.8 (7.2)	2.47	0.1179
Newborns visited within 48 hours after birth	16.5 (9.5)	9.8 (5.4)	20 (10.6)	19.9 (8.9)	2.79	0.0933
Counselled on EBF	35.3 (27.3)	17.6 (13.0)	40.8 (30.8)	47.4 (29.0)	2.24	0.1403
Newborns referred with danger signs	0.4 (0.5)	0.3 (0.3)	0.3 (0.4)	0.5 (0.7)	0.33	0.725

*SD* standard deviation, *ANOVA* Analysis of variance, *ANC* antenatal care, *EBF* exclusive breastfeeding

*P-value <0.05 statistically significant; ** data available for 4 facilities for periods 1 and 2 only and t-tests statistics computed

### Post hoc tests of variations between time periods

At both health facility and community midwifery units, there were no statistically significant differences in mean attendance for MNH services between period 1 and period 2 (*P* ≥ 0.05). However, there were statistically significant differences in mean scores for MNH services between periods 2 and 3 for 1st ANC, 4th ANC and skilled births (*P* < 0.05). At the facility level, there were statistically significant reductions in attendance for 1st ANC, 4th ANC and skilled births between the first two time periods and the third, the greatest reduction being for skilled births with a mean difference of 81.8 between period 2 and period 3. At the community midwifery units, there were statistically significant improvements in attendance for 1st ANC, 4th ANC and skilled births between the first two time periods and the third, the greatest increase being for skilled births between periods 1 and 3 (mean difference of 11.6) and periods 2 and 3 (mean difference of 11.4) (*P* < 0.0001) (Table 4).

**Table 4:** Pairwise comparison of means for MNH service utilization between groups/periods

Indicator	Groups	Mean difference	Std Error	*P*-value	95%CI	
					Lower	Upper
Facility level						
1st ANC attendance	Period 2 vs Period 1	15.1	14.3	0.553	-22.0	52.2
	Period 3 vs Period 1	-51.7	14.3	0.007*	-88.8	-14.6
	Period 3 vs Period 2	-66.8	14.3	0.001*	-103.9	-29.7
4th ANC attendance	Period 2 vs Period 1	8.4	7.5	0.515	-11.1	28.0
	Period 3 vs Period 1	-28.4	7.5	0.005*	-47.9	-8.8
	Period 3 vs Period 2	-36.8	7.5	0.001*	-56.4	-17.3
Skilled births	Period 2 vs Period 1	24.3	20.2	0.468	-28.1	76.7
	Period 3 vs Period 1	-57.4	20.2	0.032*	-109.8	-5.0
	Period 3 vs Period 2	-81.8	19.0	0.003*	-134.2	-29.3
Community midwives						
1st ANC attendance	Period 2 vs Period 1	0.4	2.5	0.983	-6.1	6.9
	Period 3 vs Period 1	8.1	2.5	0.014*	1.6	14.6
	Period 3 vs Period 2	7.7	2.5	0.021*	1.2	14.2
4th ANC attendance	Period 2 vs Period 1	-0.5	2.1	0.973	-6.0	5.1
	Period 3 vs Period 1	5.6	2.1	0.047*	0.1	11.2
	Period 3 vs Period 2	6.1	2.1	0.031*	0.5	11.7
Skilled births	Period 2 vs Period 1	0.2	1.8	0.992	-4.6	5.0
	Period 3 vs Period 1	11.6	1.8	<0.0001*	6.8	16.4
	Period 3 vs Period 2	11.4	1.8	<0.0001*	6.6	16.2

*CI* confidence interval, *ANC* antenatal care

*P-value <0.05 statistically significant

### Effect of community midwifery model on MNH service utilization

There was a statistically significant increase in the proportion of maternal and newborn health clients served by community midwives during the national strike by nurses/midwives. First and fourth ANC, skilled births and postnatal care utilization increased by 68.0, 74.5, 67.8 and 33.3 percentage points respectively (*P* ≤ 0.0001) during the implementation of the enhanced community midwifery model (Table 5).

**Table 5:** Test of differences in proportions of clients served by community midwives over time

	Pre - CMM				Post – CMM				Diff	*P*-value
	HF	CM	TOTAL (N)	Proportion (%)	HF	CM	TOTAL (N)	Proportion (%)		
1^st^ ANC	2278	74	2352	3.1	121	298	419	71.1	68.0	<0.0001*
4^th^ ANC	1218	55	1273	4.3	57	212	269	78.8	74.5	<0.0001*
Skilled births	2707	58	2765	2.1	169	393	562	69.9	67.8	<0.0001*
Postnatal care**	694	64	758	8.4	429	307	736	41.7	33.3	<0.0001*

*CMM* community midwifery model, *HF* health facility, *CM* community midwives, *ANC* antenatal care, *diff* difference in proportion (post – pre)

*P-value <0.05 statistically significant; ** calculated for 4 facility-CM dyads with data

### Maternal and newborn health outcomes

There were statistically significant differences between the three periods in the average number of babies born and discharged alive and the neonatal deaths at the facility (*P* < 0.05). On average, the number of babies discharged alive and neonatal deaths were highest in period 2 (when there was no strike) and lowest in period 3 (during the strike by nurses/midwives). However, there were no statistically significant differences in the cases of macerated and fresh stillbirths and maternal deaths between the three periods (Table 6).

**Table 6:** Kruskal – Wallis/One-way ANOVA test of differences in maternal and newborn health outcomes

Facility outcomes	Total	Period 1	Period 2	Period 3	ANOVA	*P*-value
	Mean (SD)	Mean (SD)	Mean (SD)	Mean (SD)	F-value/X^2^	
Babies discharged alive	52.2 (48.3)	63.8 (35.9)	87.2 (49.0)	5.7 (6.6)	8.49	0.0034*
Macerated stillbirths	**	61.5	69	40.5	3.084	0.2139
Fresh stillbirths	**	61.5	68.5	41	2.649	0.266
Neonatal deaths	**	54	82	35	7.651	0.0218*
Maternal deaths	**	64	54.5	52.5	0.839	0.6574

*SD* standard deviation

*P-value<0.05 statistically significant, ** values small and Kruskal-Wallis performed, X^2^ value following Kruskal-Wallis

Results from pairwise mean comparison tests showed that there was a statistically significant reduction in the average number of babies discharged alive between periods 1 and 3 (mean difference of 58.1) and periods 2 and 3 (mean difference of 81.4). Besides, there was a statistically significant reduction in neonatal deaths between periods 2 and 3 with a mean difference of 0.6 (Table 7).

**Table 7:** Pairwise mean comparison test of differences in MNH outcomes

	Groups	Mean difference	Std Error	*P*-value	95%CI (Lower, Upper)	
Babies discharged alive	Period 2 vs Period 1	23.3	20.3	0.502	-29.5	76.2
	Period 3 vs Period 1	-58.1	20.3	0.031*	-111.0	-5.2
	Period 3 vs Period 2	-81.4	20.3	0.003*	-134.3	-28.6
Neonatal deaths	Period 2 vs Period 1	0.5	0.2	0.132	-0.1	1.1
	Period 3 vs Period 1	-0.1	0.2	0.848	-0.8	0.5
	Period 3 vs Period 2	-0.6	0.2	0.049*	-1.3	0.0

*P<0.05 statistically significant

### Effect of community midwifery model on MNH outcomes

There was a statistically significant increase in the proportion of babies born and discharged alive by both the community midwives and health facilities, from 98–100% during the enhanced community midwifery model intervention (p = 0.0004). During the same period, macerated stillbirths halved from 0.7–0.36% and neonatal deaths reduced three times from 0.54–0.18% although these differences were not statistically significant. There were no statistically significant differences in the proportions of fresh stillbirths and maternal deaths following the intervention (Table 8).

**Table 8:** Pre and during/post community midwifery model intervention MNH outcomes

	Pre-CMM intervention			Post-CMM intervention			diff	*P*-value
	N	n	Proportion (%)	N	n	Proportion (%)		
Macerated stillbirths^a^	2707	19	0.70	562	2	0.36	0.35	0.1751
Fresh stillbirths^a^	2707	15	0.55	562	3	0.53	0.02	0.4764
Neonatal deaths^b^	2772	15	0.54	565	1	0.18	0.36	0.1267
Maternal deaths^a^	2707	3	0.11	562	1	0.18	-0.07	0.3394
Babies discharged alive ^b^	2772	2718	98.05	565	565	100	-1.9	0.0004*

^a^denominator (N) – total community and facility births; ^b^ denominator (N) – total community and facility live births

*P<0.05 statistically significant

## Discussion

This study assessed the effect of an enhanced community midwifery model on utilization and outcomes of maternal and newborn health services in hard-to-reach rural settings in the context of industrial actions by healthcare providers in the public sector in Kenya. Findings show that there was no statistically significant difference in the utilization of ANC, childbirth and postnatal care services between the periods when doctors were on strike and when there was no industrial action by health care providers. This can be attributed to the fact that in most low-resource settings, these services are largely provided by nurses/midwives and essentially most pregnancies and births are uneventful with around 15 percent of all pregnant women likely to develop a potentially life threatening complication that require skilled care or major obstetric interventions [42]. Secondly, the staffing levels in Kenya’s public health sector of 4,000 doctors and 47,000 nurses/midwives implies that health facilities can provide the minimum basic care by using nurses/midwives for the estimated 70–80 percent of pregnant women entering labor that are classified as low risk by the World Health Organization [42, 43]. The chronic shortage of healthcare staff in the country is similar to sub-Saharan Africa [8] and other resource-limited settings, with evidence suggesting that the shortage of skilled birth attendants is even more severe in rural compared to urban areas [4].

The findings of this study show that community midwives complemented the existing MNH service delivery systems and their role was very critical especially during the strike by nurses/midwives in the public sector. Considering that nurse/midwives are the majority providers of care during pregnancy and childbirth, their absence occasioned by a health crisis such as strikes or remoteness of locations where clients live provides an option to consider the available experienced and retired skilled health personnel to bridge the gap in service delivery under such circumstances. Evidence from Nigeria and Ghana demonstrated that strengthening community engagement and training in handling normal births and referring complications improved ANC coverage and normal births in hard-to-reach communities [44, 45].

Our findings suggest that reorientation of traditional birth attendants to BCs and community sensitizations by CHVs are important for ensuring that communities seek skilled birth attendance during pregnancy and childbirth. The findings are consistent with those of studies conducted in Nigeria and Kenya which showed that CHVs and BCs are key decision influencers and/or decision – makers at the community level regarding care-seeking for skilled maternal and newborn health services, especially in poor rural communities where home births assisted by traditional birth attendants are prevalent [21, 46]. Evidence in rural Kenyan settings shows that delivery of health messages by CHVs increases knowledge of maternal and newborn care among women in the local community and improves births under skilled care [47]. In addition, the 2017 Kenya confidential enquiry into maternal deaths reports revealed that failure to recognize danger signs (12%) and delay in deciding to refer (11%) were the most frequently identified community factors associated with maternal deaths that can be addressed by service providers at that level [27].

The significant improvements in the proportion of women who accessed skilled ANC and childbirth services from the community midwives during the nurses/midwives’ strike is crucial for achieving skilled birth attendance and improving the quality of maternal and newborn health in the country. Evidence suggests that 16 to 33 percent of all maternal deaths in developing countries could be prevented through skilled birth attendance [48]. Further, promoting skilled birth attendance is based on evidence that at least 75 percent of maternal deaths occur from late pregnancy to 48 hours following delivery [49]. In Kenya, almost half of the intrapartum fetal deaths occur in the antenatal and intrapartum period and over half (55.8 percent) of the maternal deaths occur in the intrapartum and postpartum period [27]. Our findings indicate that involvement of community midwives in the provision of these essential services contributes to averting complications that would arise from delays in seeking care. The findings suggest that supporting community midwives with regular knowledge and skills updates (through onsite mentorship and supportive supervision) and strengthening community linkages and referrals enhance their capacity to manage or refer cases with complications and thus avert deaths. It is also possible that given their experience (being retired healthcare providers), they were held in high esteem by the community, which enabled them to seamlessly integrate into the health system.

Strengthening linkages between health facilities and community health interventions, defined as a formalized connection between a health facility and the communities it serves to support improved health outcomes, is integral to improving access to and utilization of essential MNH services [50–52]. The Kenya Service Provision Assessment Survey (2010) showed that linkage between public health facilities and the communities was poor at only four percent (compared to non – governmental facilities at 30 percent) [53]. Given that up to 39 percent of pregnant women deliver at home in Kenya [26], a community midwifery model involving the provision of essential MNH services at that level is important for optimizing access to and utilization of the services [54]. However, poor access due to geographical factors (including long distances to health facilities) and limited availability of referral systems for maternity emergencies are major barriers to service uptake in hard-to-reach rural areas [53, 55]. In the rural areas of Kenya, 48 percent of people live more than 5 kilometres away from a health facility and only three percent of the facilities having a linkage with the communities they serve [53] which suggests that such areas could greatly benefit from community midwifery interventions.

Community midwives charged minimal user fees (including acceptance of other non – monetary payment) for the services provided. User fees or out-of-pocket cost with the option of non – monetary payment could be a major determinant of access to and uptake of skilled care in the community. Cost is a key deterrent to access to and utilization of skilled pregnancy and childbirth care services especially among women in rural areas [22, 56]. Our findings suggest that recognizing and supporting community midwives to be functional as key providers of care at the community level through provision of supplies, motivation and support supervision is important for consolidating the gains in utilization of MNH services in public health facilities in the country associated with the free maternity policy that took effect in 2013 [21, 56, 57]. The free maternity policy aimed to make maternity services accessible and affordable, and to reduce maternal and perinatal deaths in the country [56]. Our findings suggest that using community midwives can play an important role in strengthening the health system in low-resourced rural settings, reduce the cost of obtaining care, and contribute to achieving universal health coverage.

There were increased household newborn visitations within 48 hours after births by CHVs, identification and referral of newborns with danger signs and enhanced health education and sensitization on benefits of exclusive breastfeeding which could have contributed to the significant reductions in neonatal deaths. These findings are similar to those of systematic reviews conducted in developing countries on the effect of community health intervention packages involving training of community midwives on maternal and perinatal outcomes [58, 59]. Evidence shows that multisectoral interventions that involve the use of community midwives contribute to a reduction in maternal mortality ratios in developing countries [60]. This underscores the importance of strengthening MNH services at the community level.

### Strengths and weaknesses

Our study took advantage of an exogenous stressor (the industrial action by healthcare workers in the public sector) to demonstrate how using resources available at the community (community midwives, community health volunteers and birth companions) could improve uptake of maternal and newborn health services in times of crises or in settings with limited resources.

This study relied on data from the DHIS2 which may have weaknesses in completeness, quality and accuracy of information. In order to minimize these challenges, the project and county teams conducted regular supervision of data capture and reporting processes. The small number of community midwives involved in the project could limit the detection of statistically significant changes in the number of clients they served or maternal and newborn health outcomes attributable to their services over time.

## Conclusions

Our findings show that using resources available at the community level bridges the gap in delivery of maternal and newborn health services where there is shortage of skilled health care personnel occasioned by industrial action or limited availability of facilities. This, however, requires a collaborative approach involving local level policy makers and community-level resource persons, including community midwives and community health volunteers, to create awareness and demand, and to provide services that can be delivered at the community level. The findings underscore the importance of integrating these community-level health service providers (community midwives and health volunteers) into the primary health care system to complement service delivery according to their level of expertise, especially in low-resource settings similar to the study location.

## Data Availability

The datasets used and/or analysed during the current study are available from the corresponding author on reasonable request. The data was extracted from the Kenya Health Information System (KHIS), formerly the District Health Information System 2 (DHIS2), an open source public access system where all MOH reporting is done and requires registration credentials to access. The link to the databases used is https://hiskenya.org/dhis-web-pivot/.

## Abbreviations

ANC: Antenatal care
BCs: Birth companions
CHEWs: Community health extension workers
CHVs: Community health volunteers
CI: Confidence Interval
CMM: Community midwifery model
CMs: Community midwives
DHIS: District Health Information System
EmONC: Emergency Obstetrics and Newborn Care
FANC: Focused antenatal care
FIGO: International Federation of Gynecology and Obstetrics
ICM: International Confederation of Midwives
MOH: Ministry of Health
MPDSR: Maternal and Perinatal Death Surveillance and Response
PNC: Postnatal care
SHP: Skilled health personnel
WHO: World Health Organization
